# Beyond genes and environment: mapping biological stochasticity in aging

**DOI:** 10.1007/s11357-025-01673-y

**Published:** 2025-04-29

**Authors:** Adam J. Hruby, Gilberto Garcia, Max A. Thorwald, Caleb E. Finch, Joshua Johnson, Ryo Higuchi-Sanabria

**Affiliations:** 1 Leonard Davis School of Gerontology, University of Southern California, Los Angeles, CA 90089 USA; 2 Department of Obstetrics and Gynecology, University of Colorado-Anschutz Medical Campus, Denver, CO USA

**Keywords:** Aging, Stochasticity, Variability

## Abstract

Aging is characterized by extensive variability in the onset of morbidity and mortality, even in genetically identical populations with carefully controlled environments. This points to the important role stochasticity plays in shaping the divergent aging process between individual organisms. Here, we survey how stochastic factors at the level of molecules, cells, tissues, and organisms manifest in and impact the aging process, with a focus on the nematode *Caenorhabditis elegans*. Findings of stochasticity in *C. elegans* give additional insights for aspects of aging in the more complex settings of mammals with parallels drawn between organisms when appropriate. The emerging understanding of the stochastic contributors to longevity will enhance research strategies and medical interventions for personalized medicine.

## Introduction

A degree of variation in morbidity and mortality is a common feature of aging organisms across the tree of life. Even genetically identical individuals vary widely in lifespan, with human identical twins and nematodes (*Caenorhabditis elegans*) having similar heritability of lifespans [1–3]. Although human twins may be subject to different environmental factors, adult worms in the laboratory have negligible environmental variations because they hatch and live in the same medium. Yet, individual worms swim, feed, and oviposit at different rates, and their lifespan may differ more than threefold [4]. While individual worms have minor anatomical differences, human twins differ widely in cell numbers that may arise from stochastic variability in several processes. These can include variation in gene expression, cell migration, and proliferation during development, compounded by the acquisition of somatic mutations and oxidative damage to long-lived proteins through other stochastic processes during aging. Tissue and organ changes then shape the detectable differences between twins. Differences in lifespan are typically thought to be the result of interactions between an individual’s genome and its environment. However, the fact that isogenic laboratory animals raised in identical environments continue to display differences in lifespan is strong evidence that a third component, inputs which we term “biological stochasticity,” plays a significant role in determining variation in lifespan.

The past decade has seen a rapid advance in characterizing the molecular and cellular consequences of the aging process. The influential Hallmarks of Aging were elaborated in 2013 [5] followed by other attempts to codify aging [6], including an update to the original Hallmarks [7]. While these efforts have largely agreed upon the types of changes that occur with aging across diverse species, the cause of the great variability in lifespan within individuals of the same species has received much less attention. Lifespan heritability at the species level is below 35% for human twins, inbred flies and mice, and between nematode lines [1]. Nonetheless, the coefficients of variation in lifespan (CV_LS_) can vary widely in different species [8, 9]. For example, chimpanzees living in the wild have a CV_LS_ of 0.98 while human populations in developed countries have a CV_LS_ of approximately 0.15 [9]. More remarkably, CV_LS_ exhibit significant variation even between genetically identical individuals. Isogenic *C. elegans* [3], inbred mice [10], and identical human twins [2] all exhibit similar CV_LS_, in the range of 15 to 30% [1]. Some of the strongest experimental evidence that lifespan is an inherently variable trait has been provided by the National Institute on Aging’s *Caenorhabditis* Intervention Testing Program (CITP). Begun in 2013, the goal of the CITP is to identify compounds which robustly and reproducibly extend lifespan across three different *Caenorhabditis* species and multiple different strains, with work replicated by three separate institutions [11–13]. Lifespan data collected from more than 725,000 animals over 891 trials revealed that 58.4% of individual variation in lifespan was due to undefined factors, demonstrating the fundamentally stochastic nature of longevity [13]. The remaining variability in lifespan was due to genetic variation (25%), differences within labs (14.6%), and differences between labs (2%). The 16.6% variation found within and between labs was speculated to be caused by either stochastic factors that affect aging or uncontrollable environmental factors such as differences in atmospheric pressure, humidity, exposure to light, or temperature changes that occur outside incubators. This level of variation despite standardization of methods and reagents underscores how difficult reproducing lifespan findings can be and is important to consider when interpreting studies of how stochastic biological factors influence aging. Nevertheless, nematode lifespans exhibit nearly 60% variability despite strict controls for nearly all intrinsic cellular and genetic factors as well as environmental conditions that might affect aging, suggesting that additional factors are at work.

Studies using *C. elegans* have made significant strides in understanding the causes of stochasticity in lifespan, with several factors making *C. elegans* uniquely suitable for this task. In unstressed conditions, *C. elegans* exist as hermaphrodites, possessing the means to self-fertilize; this “selfing” ensures that genetically identical individuals are produced each generation and removes the potential for sex differences to complicate measures of stochasticity. Differences in the environment can also be easily minimized by equally distributing the bacterial food source (typically *E. coli*) and maintaining constant temperature and humidity in an environmental chamber (i.e., temperature- and humidity-controlled incubator). Lastly, *C. elegans* has been used extensively as a model for aging research [14] and much is known regarding the genetic pathways that regulate its lifespan. Taking advantage of this model of biological “standardization” allows the probing of exactly how the above-mentioned significant differences in lifespan occur and to address the hypothesis that inputs of “biological stochasticity” give rise to those differences.

In its simplest form, we define stochasticity as an event or outcome being randomly determined. While an exhaustive mathematical, philosophical, or even empirical definition of stochasticity is beyond the scope of this review, there are a few examples relevant to biology that can be used to understand stochasticity in its most primitive form: the movement of a molecule is random and cannot be predicted. Referred to as the “Wiener Process” or “Brownian Motion,” the movement of microscopic particles is random, although the contents of the environment can somewhat influence these movements by bombardment of molecules with surrounding molecules or medium. Again, a scientific model of how these random Brownian motions can impact biology and physiology is beyond the scope of our work. Instead, we use the oversimplified term “biological stochasticity” (hereafter referred to as stochasticity for ease), to define the seemingly random differences within biological organisms (inputs) that can have a measurable impact on cellular or organismal physiology (outputs; here, aging), but cannot be ascribed to differences in genetics or environment.

**Fig. 1:**
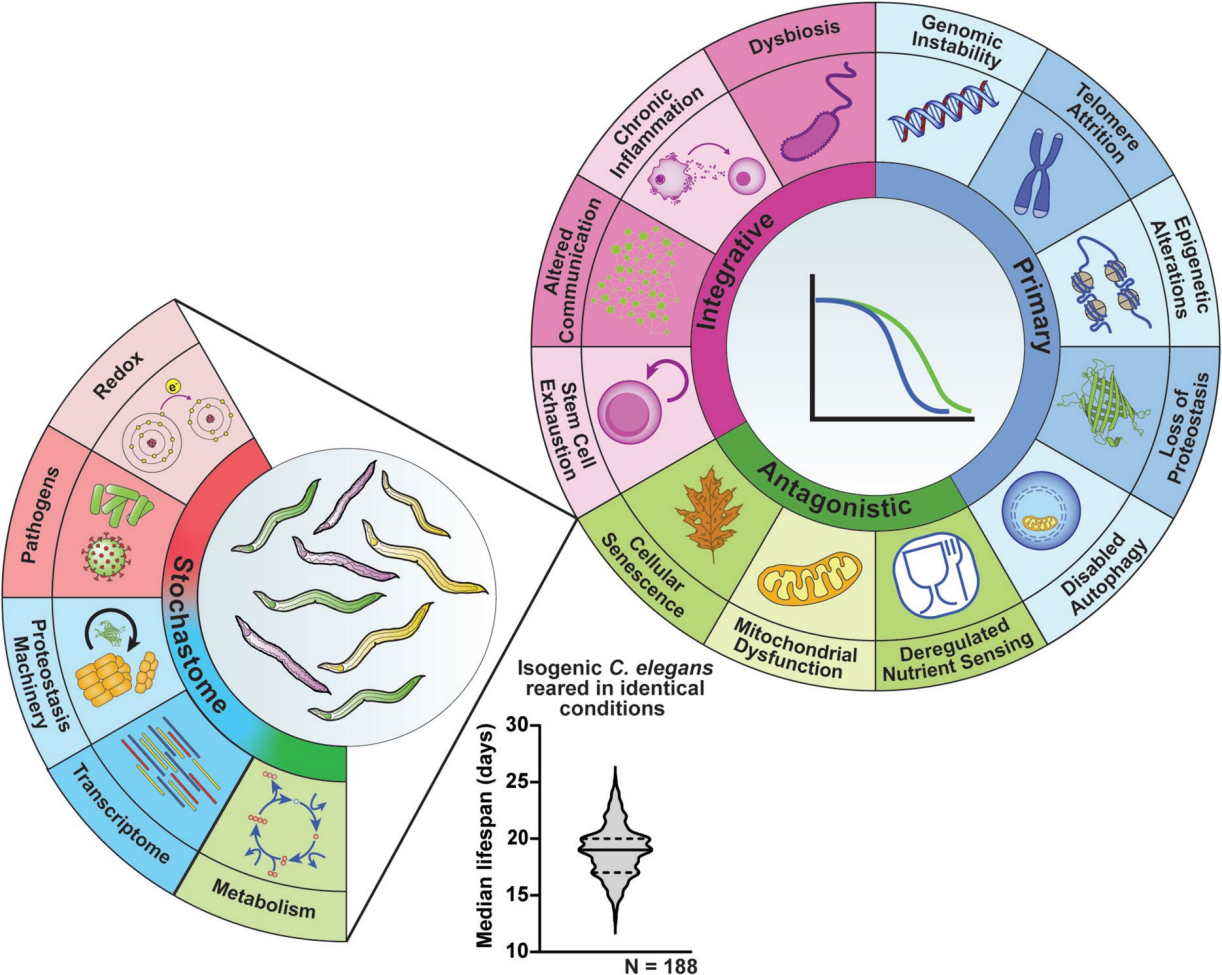
Stochasticity underlies variable outcomes in aging. Diverse molecular, cellular, and intracellular events that occur throughout the life course, here represented by the Hallmarks of Aging, manifest as declining organismal function and an increasing risk of mortality. However, the rate of decline is not uniform for all organisms of a population, even when considering variable genetic and environmental factors. This points to fundamentally stochastic events as drivers of heterogeneity in healthspan and lifespan in aging. Studies using the nematode worm *C. elegans* have experimentally demonstrated that stochastic variation in a diverse array of biological processes affect aging outcomes, suggesting stochastic events that can significantly impact aging biology are common. The violin plot represents 188 median wild-type *C. elegans* lifespans (each *N* has a sample size > 100 worms) taken from lifespan assays performed by the same research group using wild-type *C. elegans* reared under identical conditions. This plot shows the variability of lifespans across experiments even in virtually identical conditions

We assert that stochastic events play a significant role in determining aging outcomes, highlighting studies performed in *C. elegans* that elucidate sources of this variation. We refer to this collection of stochastic factors as the “stochastome” (Fig. 1). Stochastic factors that influence aging can be determined by analyzing cell and molecular variations in *C. elegans* given that they are genetically identical and have identical cell numbers and minimal DNA mutation burden [15]. It is important to highlight the limitations of this review upfront: our oversimplification of the term “biological stochasticity” is minimized to the seemingly random (i.e., cannot be explained through genes or environment) factors that contribute to aging. However, we say “seemingly” random, as it is entirely likely that the mechanistic input of gene or environment has not yet been established, rather than the factor being truly stochastic. Rather than using complex statistical or mathematical analysis/modeling, our analysis of the scale and levels of stochasticity in nematodes provides an introductory definition of stochasticity to provide an entry point into the field, as well as a template for analyzing the apparently far greater human developmental stochasticity that contributes to variability in aging. Stochasticity that arises from cell migration during development and gene expression variations, as well as genomic and proteomic damage over time, are all potential contributors. Determining what processes exhibit stochasticity in *C. elegans* as well as humans holds the promise of shedding new light on the molecular and cellular drivers of the aging process [16, 17].

## Stochasticity in aging outcomes in *C. elegans* and mammals

Stochastic features of aging are discussed at all levels from the organismal to the molecular. Each section summarizes domains of stochasticity in *C. elegans* and other animal models where available. Stochastic processes that lead to variable aging outcomes appear to be conserved between organisms, including humans, albeit with certain limitations.

### Organismal health

**Fig. 2:**
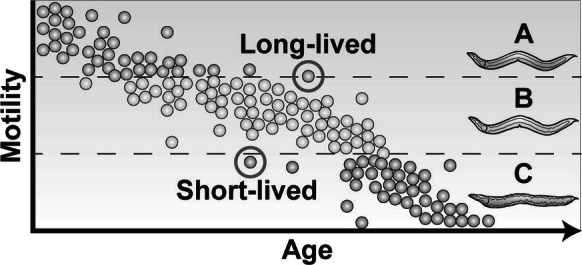
Characteristic age-related locomotor decline in an isogenic population of *C. elegans*. Animals are divided into three behavioral classes: class A move continuously in sinusoidal motions, class B move only when prodded, and class C only move the head or tail when prodded. *C. elegans* progress irreversibly through these classes with age, with almost all animals reaching class C before death. An age-matched isogenic population of animals raised in identical environments displays a heterogenous distribution of class. The behavioral class of an individual animal serves as a better predictor of mortality than age itself, suggesting stochastic processes factor strongly in how an individual ages. Animals that remain in class A longer are generally longer-lived while those that enter class C earlier are short-lived

The prominent role of stochastic processes in *C. elegans* lifespan was largely identified during efforts to characterize the pathobiology of aging at the cell and tissue level. In their seminal study, Herndon et al. [18] describe three distinct classes of locomotory behavior identified in an isogenic population of middle-aged to aged *C. elegans*. Class A animals continually move in sinusoidal motion; class B animals only move when prodded, and class C animals do not move when prodded, only displaying head or tail movement. With age, animals were found to progress from class A to C, with class acting as a better predictor than chronological age for mortality. Importantly, this rate of behavioral decline varied widely across isogenic populations raised in identical environments, strongly indicating the important contribution stochasticity plays during age-related locomotor/behavioral decline (Fig. 2).

There were two major competing hypotheses to define the variable rate in locomotor behavior during aging. First, that individual animals can follow a different aging trajectory defined by individual-level differences in the transcriptome and proteome, and their subsequent effects on organismal health. The second hypothesis posited that all animals follow the same aging trajectory, but the rate at which they move through the common trajectory differs. Single-worm proteomic analysis of similarly aged animals within different locomotor classes revealed that animals followed a similar aging trajectory at the proteomic level but at different rates [19]. Proteomic analysis from day 1 to day 10 of adulthood identified an aging proteomic profile in worms that exhibited the largest changes from day 1 through day 5, less dramatic changes between day 6 through day 8, and changes that generally plateaued between day 9 and 10. Class A, B, and C animals all followed this same trajectory of proteomic changes and differed primarily in the rate they moved through these pseudotimes. When looking at specific differences in individual proteins between all three classes, class A and B were nearly indistinguishable, although class C animals displayed a few significant differences, including higher levels of lysosomal proteins and specific chaperones, and lower levels of vitellogenins [19]. These data provide support for the second hypothesis and the view that stochastic processes at the cellular and subcellular level play a significant role in determining the course of age-related locomotor decline.

New approaches to investigating individual differences in organismal health have strengthened evidence for stochastic onset of age-related functional decline. Zhang et al. [20] followed individual isogenic animals in identical environments throughout aging and assessed health through a variety of metrics, including movement. They found that, while all animals begin adulthood with the same level of health, the rate each individual proceeds through age-related decline differs. In addition, shorter-lived animals were found to live a smaller proportion of their lives in poorer health than long-lived animals and died in a more phenotypically healthy state, suggesting a qualitative difference in aging dynamics. This contrasts with a more recent study which made use of high-resolution video of individual worm locomotion to identify 896 morphological, postural, or behavioral features that correlate with age [21]. Segregation of animals into short-, medium-, and long-lived groups demonstrated that while shorter-lived animals progressed faster through this phenotypic space, the character of the phenotypic changes remained scaled to an individual’s lifespan.

Statzer et al. [22] developed a microfluidic device to measure maximum muscle strength and dynamic power in *C. elegans* using acoustophoretic force fields. Heterogeneity in muscle strength between individual animals was found to increase with age until day 14 of adulthood after which a reduction in heterogeneity was observed. This study also measured the onset of morbidity, defined as the point at which an individual displays slow or no voluntary movement, finding again vast variation between individuals. A similar level of variation was observed between wild-type and long-lived strains including genetic models of caloric restriction (*eat-2(ad1116)*) and reduced insulin/IGF-1 signaling (*daf-2(e1368)* and *daf-2(e1370)*), with a notable exception being the germlineless mutant *glp-1(e2141)*. *glp-1(e2141)* animals showed significantly less variation in the onset of morbidity, corroborating recent findings that the germline is responsible for roughly half of individual variation in lifespan [23], discussed in section “Transcription” below.

In addition to indicators of locomotory behavior and muscle health, a wide array of other phenotypes has been shown to predict lifespan in a genetically identical population of *C. elegans* grown in identical environments. Predictors of longer lifespan include larger size and better maintenance of body length with age, less accumulation of autofluorescent age pigments, and a less “decrepit” appearance in brightfield images as measured through image texture [20, 21]. Combining these three factors with a score of movement following stimulation with green light predicts 62% of lifespan variability across a homogenous population [21]. These broad indicators of health suggest differences in tissue maintenance, autophagocytic ability, and sarcopenia between individuals in a population that are probable downstream reflections of stochastic events occurring at the cellular and molecular level throughout aging.

### Organs and tissues—the nervous system

The rate of aging also differs considerably across tissue types. Using a combination of electron microscopy and tissue-specific promoter-controlled GFP reporters, the nervous system showed no appreciable physical deterioration with age, even in class C worms [18]. In contrast, worm muscle exhibited considerable decline with age, shown by a loss of GFP expression in body wall muscle nuclei and increased disorganization of sarcomere structure. The manifestation of these phenomena was again stochastic between isogenic individuals, with time differences in nuclear GFP expression first evident at day 9 of adulthood and both pharyngeal morphology and myofibril structure displaying stochastic decline. Stochasticity in age-related decline was also evident in the intestine and hypodermis. While no structural decline in the nervous system was observed with normal aging, more recent studies have demonstrated age-related stochastic changes in the nervous system. A deterioration of synapse integrity occurs with age and is more common in class C animals, and aged neurons exhibit increased neurite branching [24]. Both observations were found to be stochastic in nature. Neuron activity is also altered with age as the AWC sensory neuron and AIA interneuron in aged *C. elegans* undergo spontaneous and stochastic activation [25]. This hyperactivity contributes to age-related decline in thermotaxis behavior, suggesting that stochastic neuronal signaling may contribute to individual differences in aging outcomes. Lastly, studies in *C. elegans* have found that pan-neuronal expression of polyglutamine repeats characteristic of several neurodegenerative diseases can recapitulate aspects of aging, most notably a severe deficit in mobility [26].

In contrast to mammals, the structure of the nervous system in *C. elegans* has long been considered essentially invariant [27]. Nonetheless, recent efforts to map all the chemical and electrical synapses of the *C. elegans* nervous system, the connectome, have revealed variation between the left–right homologous neuron pairs and their targets [28], which are assumed to be equivalent. This suggests that similar variation likely exists between individuals. One example of a stochastic effect on lifespan derived from altering neural function comes from a recent study of heat shock factor 1 (*hsf-1*) overexpression in the four cephalic sheath glia of *C. elegans*, analogous to mammalian astrocytes. *hsf-1* overexpression results in a bimodal population consisting of long-lived and standard-lived worms, which depends on peripheral activity of *hsf-1* and *daf-16* [29]. Although no further investigation was made into the source of this bimodal distribution, it suggests that an underlying stochastic element plays a major role in determining how an individual animal responds to neural perturbations.

While these studies highlight the exciting potential of stochasticity of the nervous system contributing to variability in aging, there are important differences in *C. elegans* neurons and mammalian brains, particularly during development. In mammals, brain development begins with formation of the neural tube in the first trimester. Glial cells in the anterior tube proliferate and migrate, giving rise to future neurons of the cerebral cortex formed in the second trimester. While developmental programs establish right-left asymmetry, imprecision in cell migration adds a stochastic component to cerebral cortex development. A large human study using UK Biobank brain samples found that most brain asymmetries have a low (~10%) heritability, suggesting that variation between individuals is likely due to developmental stochasticity or environmental factors [30]. *C. elegans* also display a small degree of stochastic left–right neuronal asymmetry. One of either the left or right AWC olfactory neurons develops into the AWC^ON^ type while the other develops into AWC^OFF^, each expressing different chemoreceptor genes to detect separate odorants [31, 32]. This outcome is driven by differences in calcium between the two cells with lower levels of calcium leading to the AWC^ON^ identity and greater to AWC^OFF^.

The study of diversity in brain aging is important as many neuropathologies exhibit wide variability across individuals. Alzheimer’s disease (AD) is characterized by a progressive accumulation of extracellular amyloid aggregates and intracellular hyperphosphorylated tau tangles, neuronal death, and failing cognitive capacity which eventually leads to death. There exists extensive variation in disease progression between AD patients [33], owing to a host of genetic and environmental factors, as well as probable stochastic contributors.

Due to the heterogeneity in genetic, environmental, and experimental variables contributing to AD, studying the contribution stochastic processes play in AD pathology in humans is challenging, highlighting the need for reproducible, simplified models like *C. elegans*. However, even when controlling for genetic and environmental variables in *C. elegans* models of proteostatic diseases, stochastic variation in pathology and functional decline is still observed. For example, in transgenic *C. elegans* expressing the human amyloid beta peptide Aβ_1–42_ in neurons or body wall muscle, amyloid beta aggregation impairs fecundity, motor coordination, and shortens lifespan [34]. While total Aβ aggregation increased with age across *C. elegans* populations, notable variation was found in the amount of aggregation between individual worms. Interestingly, middle-aged worms expressing Aβ_1–42_ in the body wall muscle, but not neurons, exhibited a bimodal population with one group showing minor accumulation and the other greater accumulation [34], suggesting that there exists differences between cell types in stochastic protein aggregation.

*C. elegans* have also been used as models for other proteostatic diseases such as Huntington’s disease, in which the aggregation of polyglutamine repeats demonstrates stochasticity. Genetically identical animals expressing 40 glutamine repeats (Q40) in the body wall muscle display significant variation in the extent of aggregation, varying from 5 to over 140 aggregates despite similar expression levels [35]. In addition to worm-to-worm variability, which body wall muscle cells ultimately developed Q40 aggregates also varied. The appearance of the first Q40 aggregate when Q40 is expressed in *C. elegans* muscle cells, each of which is physiologically identical, is entirely random with no spatial relationship observed for subsequent aggregation in other cells [36]. This suggests the initiating nucleation event forming an aggregate occurs independently in each cell, mirroring similar findings of the stochastic nature of protein aggregation initiation made in vitro [37] and in cell culture [38]. In addition to both transgenic Aβ_1–42_ and polyQ models, it is important to note that normal aging in *C. elegans* is characterized by a loss of proteostatic capacity, occurring within 24 hours of adulthood at the onset of reproduction [39, 40]. Altogether, these data demonstrate that the use of transgenic *C. elegans* models can provide insight into the nature of stochastic events at the protein level in the context of human disease.

### Development and reproduction

Development in *C. elegans* is a tightly regulated process wherein the timing and patterning of cell division were initially reported to be nearly identical across individuals [41]. However, subsequent investigations revealed that some level of variation arises during development. For example, germline gene expression — both oogenesis- and spermatogenesis-related genes — has inter-individual differences during development [42]. Moreover, this study found variable development and growth rates across genetically identical offspring, with a key driving factor of this inter-individual variability being the age of the mother. Young mothers produced progeny that were impaired for numerous characteristics including size at hatching, starvation resistance, fecundity, and rate of development, although no difference in progeny lifespan was observed. These differences are thought to be largely due to vitellogenin provisioning, as vitellogenin levels increase dramatically in progeny born from mothers of day 1 to day 3 of adulthood, and yolk depletion in mothers is sufficient to induce defects in progeny development. Beside these differences between wild-type animals, stochastic variation in gene expression during development can cause the incomplete penetrance of certain mutations such as those in *skn-1* [43]. In addition, the fate of several cells during *C. elegans* development is determined in a stochastic manner due to variation in the timing of key signaling pathways [44, 45]. In both *C. elegans* and zebrafish development, the activity of the cyclin-dependent kinases in cycling cells serves as a predictor of whether a cell in G1 phase will proceed to mitosis or enter quiescence [46]. This is even the case in the vulval precursor cell known as the D cell which, while considered invariant, can stochastically divide under high temperature conditions. D cells with high CDK activity were found to re-enter the cell cycle while those with low CDK activity committed to quiescence, suggesting stochasticity in CDK activity influences cell fate decisions. The ultimate contribution developmental stochasticity plays in determining the lifespan of a single worm has yet to be fully explored, although as noted below, stochastic events in redox state during development can drive different outcomes in aging [47]. The development of other animals from the fly to humans is more complex than *C. elegans* for many reasons, including major stochasticity in reproductive organs.

Another important factor in developmental stochasticity that is challenging to study in *C. elegans* is reproductive stochasticity. A *C. elegans* hermaphrodite produces several hundred sperm during development before irreversibly switching to oogenesis, wherein over 1000 oocytes are produced de novo from a stem cell niche during the rest of the lifespan. Thus, reproductive capacity in *C. elegans* is generally sperm limited, as mating with males can increase the reproductive duration of hermaphrodites [48]. However, in mice and humans, the reduction in oocyte numbers during chronological aging occurs in an exponential decay pattern [49, 50]. In humans, ovulatory cycles cease when the ovarian pool falls below a threshold of around 1000 oocytes [49, 51], bringing about menopause, a state unique to humans and a few other mammalian species [52, 53]. Recent work has interrogated the threshold number of follicles at which cycles can no longer continue, suggesting that stochastic control results in an ever more irregular supply of growing follicles with aging. Once stochastic “gaps” in time — where no growing follicles are available —reach 12 days, menstrual cycles cannot continue [54]. This suggests that a major determinant of the timing of menopause is the number of primordial follicles that an individual's ovaries contain at birth.

Vertebrates such as humans and mice vary widely across individuals in terms of the number of primordial follicles that are present at birth [16, 49, 50]. At 3 months of age, inbred mice can vary fivefold in oocyte number. The origin of variation in oocyte number may itself also involve stochastic processes. Primordial germ cells (PGCs) arise in extra-embryonic tissues of the yolk sac and proliferate while migrating through the embryo toward and into the future ovary. PGC migration is a complex process mediated by interactions with diverse cell types and signaling pathways [55]. Not all PGCs successfully complete this process, with approximately 5% of PGCs being eliminated through errors in migration and subsequent apoptotic cell death [55, 56], although PGC loss has not been explicitly quantified for stochasticity. PGC migration and oocyte selection for elimination are just a few examples of developmental processes that could account for variation in at-birth oocyte numbers, as PGCs continue to face rounds of quality control and apoptosis [57]. The number of follicles “endowed” at birth then engage in an exponential decay curve during and beyond the reproductive years until menopause is reached [54].

## Stochastic sources of variability in lifespan

Here, the diverse molecular and cellular events which ultimately lead to stochastic variation in lifespan are explored.

### Transcription

The application of gene expression profiling techniques like microarrays and later single-worm transcriptomics allowed for the investigation of stochasticity in aging at the transcriptional level. Gene expression profiles based on full genome microarrays of isogenic *C. elegans* show separate clustering both by chronological age as well as behavioral class [58]. This suggests that an individual worm’s transcriptional profile is not only based upon chronological age, but also upon stochastic declines in behavior. For *C. elegans*, approximately 30% of all genes were found to change expression from day 4 to day 24 of adulthood as animals progressed from class A to class C. A total of 1122 genes increased from class A to C while 432 decreased; however, no single-gene candidates were identified that significantly contribute to predicting an individual’s chronological age. Despite this, several gene pathways increased in expression with age, including those involved in cell death, mitochondrial function, and collagen metabolism [58]. More recently, proteome heterogeneity was shown to increase during aging, with proteins related to DNA replication, cell death, and stress responses showing the most variability [19].

MicroRNAs (miRNAs) are non-coding RNAs that serve to repress translation of mRNA. Several studies have found that the expression level of certain miRNAs can predict lifespan in individual *C. elegans*, acting as aging biomarkers. *mir-71*, *mir-246*, and *mir-239* were the first miRNAs found to correlate with lifespan variability [59]. Expression of these miRNAs as assessed through a transgenic GFP reporter driven by their respective promoters was shown to increase with age, with *mir-71* and *mir-246* plateauing and then declining around day 6 of adulthood. Animals with higher or longer lasting expression of *mir-71::GFP* live longer, predicting 35% of variation in lifespan; animals with more slowly plateauing levels of *mir- 246::GFP* live longer, predicting 20% of variation in lifespan; *mir-239::GFP* expression dynamics account for 10% of lifespan variability, with longer lifespan observed in animals with lower expression. *mir-71* is known to regulate the insulin/IGF- 1-like signaling pathway [60], and its expression level in the *daf-16(mu86)* background which lacks an important effector of this pathway no longer predicts individual lifespan. This suggests that stochastic variation in *mir-71* may modulate insulin/IGF-1-like signaling and thereby affect lifespan. A follow-up study screened 22 miRNA::GFP reporters for ability to predict lifespan [61]. Of these, 10 genes (*lin-4*, *mir-47*, *mir-60*, *mir-85*, *mir-90*, *mir-228*, *mir-240–786*, *mir-243*, *mir-246*, and *mir-793*) were able to predict lifespan with a joint correlation coefficient (*R*^2^) value ranging from 0.166 to 0.328.

An additional source of individual variation in an isogenic population is random monoallelic expression (RMAE) in which only a single allele is actively expressed with the other being silent. RMAE is relatively common with greater than 5% of autosomal genes being subject to RMAE in humans [62] and more than 10% in mice [63]. This can lead to manifestation of recessive traits or escape from dominant mutations. In *C. elegans*, use of a dual fluorescent reporter system expressed at the same locus revealed that two H3K9 histone methyltransferases regulate RMAE. MET- 2 acts as a negative regulator and SET- 25 as a positive regulator [64]. Although effects on inter-individual lifespan variance were not measured, *met-2* mutants did display a decreased lifespan and *set-25* mutants lived slightly longer than wild type, suggesting RMAE may influence lifespan.

Recent work utilizing single-worm transcriptomic sequencing across thousands of animals provided unparalleled resolution of the non-genetic drivers of lifespan variance [23]. Transcriptomic analysis of wild-type animals from day 1 to day 8 of adulthood showed that nearly half (47%) of the transcriptome displays age-associated increases in gene expression variance. Quantification of total mRNA in the soma versus the germline revealed a close correlation between somatic and germline transcripts which declined during aging. Artificially inducing this loss of correlation through germline-specific RNA interference of RNA polymerase II subunits recapitulated age-related increases of transcriptional variance, with similar results found in germline-ablated animals. In addition, lifespan variance in germline-ablated mutants was half that of wild-type animals. Regression modeling of transcriptomic data from these germline-ablated mutants found that an intact germline is responsible for 58% of lifespan variation in wild-type animals. The authors conclude that the largest contributor to non-genetic lifespan variance is a decoupling of somatic and germline mRNA. To identify the mechanisms underlying non-genetic lifespan variation, a large-scale screen was performed using both genetic and environmental interventions, finding 40 hits that each explain at least 10% of gene expression variation. These hits included genes of diverse function but unexpectedly did not include a non-pathogenic food source contrary to previous findings [65]. Despite their functional diversity, analysis of principal components revealed that all 40 hits converge on modulating germline-to-soma mRNA content with experimental evidence demonstrating significant reductions in lifespan variation via RNA interference knockdown of 14 of these hits. While decoupling of mRNA content was found to be responsible for half of lifespan variation, the other half remained unexplained. Analysis of transcriptomic data from day 8 animals while controlling for individual variation in somatic and germline mRNA content identified 16 gene co-expression groups which may underlie the remaining 50% of variation. Interestingly, 30% of the genes in these groups are conserved between *C. elegans* and humans, suggesting potential mediators of stochasticity of lifespan in other organisms.

### Proteostatic machinery

**Fig. 3:**
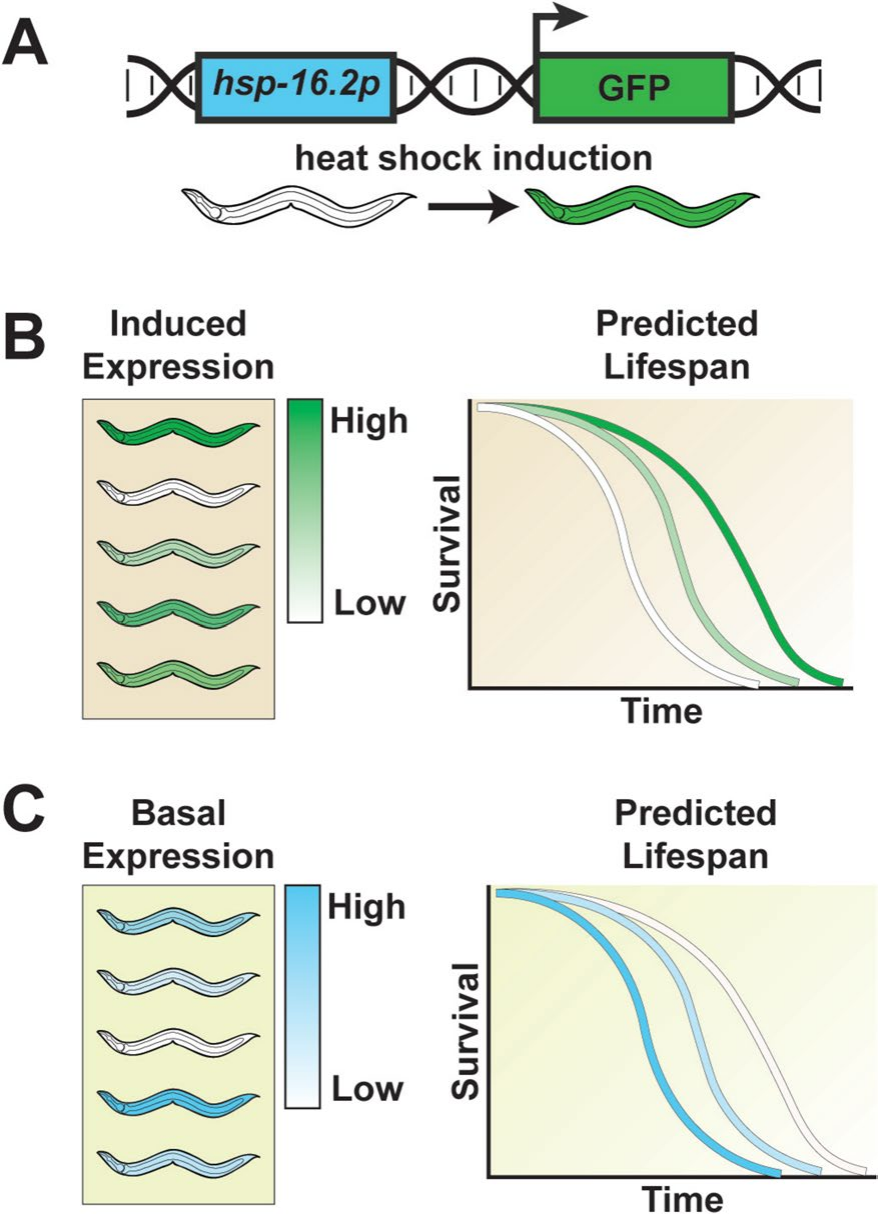
Induction of *hsp-16.2p::GFP* reporter predicts lifespan in *C. elegans*. **A** *hsp-16.2p::GFP* is a transcriptional reporter used to measure the level of the heat-shock response (HSR). When the *hsp-16.2* promoter is active (i.e., HSR is on), then GFP expression levels will be higher. *hsp-16.2p::GFP* expression serves as a strong predictor of lifespan. **B** One hour of heat shock during day 1 of adulthood results in a robust increase in *hsp-16.2p::GFP* levels (depicted here in green, where brighter shades of green indicate higher *hsp-16.2p::GFP* expression and lighter shades closer to white indicate lower expression). However, in a population of isogenic *C. elegans*, the induction is variable between individuals. Sorting these worms by the level of induced *hsp-16.2p::GFP* expression predicts remaining lifespan, with higher expression associated with longer life. **C** Basal expression levels of *hsp-16.2p::GFP* (depicted here in blue, where brighter shades of blue indicate higher *hsp- 16.2p::GFP* expression and lighter shades closer to white indicate lower expression) are typically marginal, although strong basal *hsp-16.2p::GFP* expression in adulthood can occur and is predictive of reduced lifespan

Initial efforts to characterize a single-gene predictor of aging in *C. elegans* centered on chaperones. Expression of the small heat shock protein HSP-16.2 was the first single-gene predictor of longevity established [66, 67]. *hsp-16.2* is typically only expressed in response to heat stress and acts downstream of the master heat shock response regulator HSF-1. Providing 1 hour of heat shock to an isogenic population of adult transgenic worms expressing a GFP reporter downstream of the *hsp-16.2* promoter (Fig. 3A) resulted in significant variation in *hsp-16.2p::GFP* expression. Sorting worms by high, medium, and low expression several hours after heat shock served to predict remaining lifespan, with the difference in lifespan between worms expressing high levels of *hsp-16.2p::GFP* versus low levels being as great as 10 to 15 days, in favor of individuals with high expression (Fig. 3B). In addition, higher expression of *hsp- 16.2p::GFP* also correlated with greater thermotolerance.

Expression of *hsp-16.2* itself is not thought to be the primary determinant of lifespan, as *hsp-16.2* overexpression only results in minor extension in lifespan [68]. Importantly, while this initial study failed to find a heritable component to *hsp-16.2p::GFP* expression, a follow-up study by the same group did report a significant incidence of brighter progeny from worms that expressed high levels as opposed to low levels, indicating that an epigenetic component is at play [69]. Speculative causes of stochasticity in *hsp16.2* expression included variation in protein production machinery or chance variation in the amount of HSP-16.2 present at the time of heat shock.

Later work sought to experimentally determine the source of *hsp-16.2* variation across individual cells and organisms. Burnaevskiy et al. [70] made use of fluorescent reporters controlled by the same or different promoters to tease apart the contribution that “noise” in gene expression, the activity of specific signaling pathways, or differences in protein dosage owing to the chaperone activity of *hsp-16.2* play in cell-to-cell variation of *hsp-16.2* expression in the intestine. The authors find that intrinsic gene expression noise and signaling noise play only a minor role in *hsp- 16.2* expression; instead, the capacity for the cell to both produce proteins and reduce protein turnover was the main source of variation. Previous work has shown that global heat shock response depends upon the activity of a pair of thermosensory neurons, the AFD neurons [71], and that heat shock response factors play a role in regulating the insulin/IGF-1-like signaling pathway [72]. Furthermore, AFD activity and the insulin/IGF-1-like signaling pathway both modulate the variability of *hsp-16.2* expression in response to heat shock [73], as well as the expression of other chaperones [74]. The authors suggest that differences in AFD neuron activity in response to environmental perception ultimately drive differences in chaperone expression and thus gene dosage owing to the ability of chaperones to promote protein production and decrease turnover [70].

Although canonically *hsp-16.2* is not thought to be expressed in the absence of heat stress, stochastic basal expression does occur and can be predictive of lifespan [75] (Fig. 3C). Beginning as early as day 4 of adulthood, some animals begin to robustly express *hsp-16.2p::GFP*. With age, the percentage of these worms increases, and the rate of this increase is greater at 25 °C than 20 °C, a condition known to reduce lifespan. Day 4 animals sorted into the top 1% or 10% most bright are significantly shorter lived than those randomly selected. Indeed, almost all animals showed *hsp-16.2p::GFP* expression prior to death, and this phenotype was never found to reverse. The authors speculate that aberrant *hsp-16.2* expression occurs due to a collapse of proteostatic capability indicative of a crisis state. Supporting this view, worms highly expressing *hsp-16.2p::GFP* had a significantly greater accumulation of insoluble protein. Importantly, subsequent proteomics analysis verified these claims, as class C animals with the lowest motility and closest to death displayed elevated protein levels of HSP-16.2 and HSP-16.48 compared to healthier class A and B animals of the same age [19].

Outside of *C. elegans*, changes in heat shock protein expression have also been used as a biomarker of aging in *Drosophila* and are implicated in human disease. In *Drosophila*, a greater abundance of HSP expression in response to heat shock in old flies was among the earliest reported differences in age-related gene expression [76]. Even in unstressed conditions, aged *Drosophila* exhibit greater expression of Hsps [77]. Use of fluorescently tagged *hsp22* and *hsp70* reporter strains has shown that *hsp* expression can serve as a biomarker of mortality, with an increase in HSP expression negatively correlated with survival under normal aging, oxidative stress, and heat stress [78]. Interestingly, a spike in *hsp22* and *hsp70* induction occurs in the hours preceding death [79], reminiscent of the sporadic activation of *hsp- 16.2* found preceding death in *C. elegans* [75]. Inducing overexpression of several different HSPs increases lifespan as well as stress resistance [77], suggesting the increase with age may be an effort to compensate for increased proteostatic stress.

In mammals, alteration in the expression and activity of HSPs has been implicated in various age-related diseases. Overexpression of HSPs is commonly observed in cancer where they are involved in a host of oncogenic activities including suppressing apoptosis, promoting angiogenesis, aiding proliferation, and supporting invasion and metastasis [80, 81]. During normal brain aging, different regions of the brain show either an increase or decrease in the expression of HSPs, frustrating attempts at broad generalizations regarding HSP expression in aging [82]. In neurodegenerative diseases, like Alzheimer’s disease and Parkinson’s disease, HSP expression generally increases, likely as a way to combat the toxic effects of protein misfolding, although exceptions also exist [82]. However, attempts to use genome-wide association studies to find associations between single-nucleotide polymorphisms in HSPs and onset of Parkinson’s disease [83] and Alzheimer’s disease [84] have not been met with success. Whether changes in HSP activity in mammals are a source of stochasticity in aging outcomes remains an open question that warrants further investigation.

### Redox homeostasis and pathogen infection

While proteostatic stress in late life may play a role in mortality, there is also evidence that the experience of stress during development leads to molecular events that promote longevity. An isogenic population of *C. elegans* expressing a redox state reporter showed considerable variance in redox state at larval stage 2 (L2) [47]. Interestingly, L2 larvae exhibiting a more oxidized state (L2^ox^) had an average of 18% extension in median lifespan compared to those of the mean redox state, in addition to improved survival after heat shock and oxidative stress. Basal transcript levels of heat shock response and oxidative stress response genes were no different in L2^ox^ animals compared to those with a more reduced state (L2^red^); however, after heat shock, L2^ox^ animals showed a greater induction of *hsp-1* and *hsp-16.2* expression. These changes in expression were found to be caused by a redox-induced thiol modification to the SET domain of the COMPASS complex, which regulates global H3K4 trimethylation. This reduces COMPASS complex activity and thus overall H3K4 trimethylation. The ultimate cause of the difference in redox state between individual developing *C. elegans* was not uncovered, although the authors hypothesize that the apparent hormesis-like exposure to an oxidative endogenous state during development may serve to create a subpopulation of more stress-tolerant individuals, aiding survival.

Sánchez-Blanco and Kim [65] also identified a potential link from redox homeostasis to stochasticity: the expression of *sod-3 *— which encodes a superoxide dismutase important for ROS scavenging — at day 9 of adulthood displayed a significant predictive power for remaining lifespan, with the top 50% of expressors living 22% longer on average than the lowest 50%. It is important to note that *sod-3* activity itself was not responsible for lifespan extension as *sod-3* overexpression did not affect longevity. In the laboratory, *E. coli* is typically used as a food source for *C. elegans* and is mildly pathogenic. Using *sod-3p::GFP* expression as a biomarker for aging, the authors found that reducing the pathogenicity of the food source by either irradiating *E. coli* or using a less pathogenic species of bacteria reduced the correlation between *sod-3* expression and remaining lifespan as well as its variability in expression. This correlation depended upon functional *daf-16*, which encodes a FOXO transcription factor that directly regulates *sod-3* expression as part of the insulin/IGF-1-like signaling pathway [85].

Importantly, this pathway also plays a role in mediating the innate immune response [86]. The intestine acts as the major site for pathogen response [87] and also serves as the primary site of *daf-16* regulation [88]. Interestingly, only intestinal *sod-3* expression, not head expression, was predictive of lifespan. Altogether, the authors hypothesize that variable response to pathogenicity of food results in differences in *daf-16* activity and thereby *sod-3* expression in the intestine, with increased *daf-16* activity resulting in longer lifespan. Although informative, this study did not identify what may underly this varied response to pathogenicity and suggest noise in expression of *daf-16* as a hypothesis. Additional evidence exists that host–pathogen interactions are a source of stochasticity in aging, as the amount of living bacteria in the intestine of day 3 adult worms served as a weak predictor of remaining lifespan [89]. However, more recent studies have cast some doubt on the importance pathogenicity of food plays in lifespan variance, finding that growth on irradiated *E. coli*, while increasing lifespan, did not affect inter-individual variance in lifespan [23, 90].

Considering that mortality in *C. elegans* is primarily due to bacterial infection [91], it is not surprising that several factors that may influence stochasticity are also correlated with changes in pathogenicity of the bacterial food source. In fact, many lifespan extension paradigms impact death associated with bacterial infection and swelling of the pharynx. For example, mutations in *eat-2* which decrease pharyngeal pumping, and thus reduce bacterial load in the pharynx, reduces death associated with pathogenic infection of the pharynx [91]. In addition, increased permeability of the intestine due to alterations in actin dynamics with age leads to bacterial colonization, disease, and mortality [92]. Although not explicitly investigated, several groups have reported stochastic variation in the onset of intestinal colonization beginning as early as day 3 of adulthood [92] and increasing variation with age [93, 94]. Thus, differences in bacterial infection both in the pharynx and the gut can impact mortality and potentially contribute to the stochasticity found in *C. elegans* lifespan. Whether the underlying cause of this variation is due to a stochastic decline in the cytoskeletal network is an attractive hypothesis but has yet to be tested outright.

Considering the contribution of pathogen infection to aging and age-related death, one major challenge in *C. elegans* aging studies is understanding the mechanisms of calorie restriction. Although it is clear that calorie restriction is beneficial to longevity [95], it is not possible to separate the metabolic benefits and the decrease in pathogen burden and infection-related mortality in the nematode model. Rather, it is likely that both contribute, which is ultimately why calorie-restriction related longevity paradigms tend to have the most positive impacts on longevity. Considering the dramatic effect of calorie restriction on *C. elegans* lifespan, an obvious question is whether metabolic differences can drive variability in calorie consumption and impact lifespan stochasticity in worms. In fact, the accumulation of lipid droplets was recently identified as another predictor of lifespan [96]. Sorting young and middle-aged individuals by those containing high or low numbers of lipid droplets proved successful in predicting lifespan, with individuals containing more lipid droplets at middle age living on average 33% longer than those with low numbers. The authors argue that higher levels of unsaturated fatty acids could protect lipids from the accumulation of toxic oxidation during aging, but whether these differences in lipid droplets relate to variability in metabolism, food consumption, or calorie content were not addressed. Further studies to investigate the impact of metabolic variability on stochasticity in aging are an important direction for future work.

## Conclusion

As is evident from the examples presented here, stochastic variation is observed at many levels of biology. The aging process, being affected by the interactions between genes and environment that occur throughout an organism’s life, is undoubtedly impacted by these pervasive stochastic elements. Thus, stochasticity, in addition to genetics and the environment, must be considered if one is to obtain a complete picture of what underlies aging. The studies presented here shed light on how *C. elegans *— with a precise control on genetics and environment — can inform the way in which stochastic processes contribute to aging, health, and disease, and how these findings can be translated into understanding of human aging.

An important future direction for this field is further elucidation of what mechanistically drives stochasticity in aging. In *C. elegans*, environmental and genetic variability can be ruled out, and thus one area of possibility is epigenetic variability, which can drive stochasticity in both the transcriptome and proteome. Indeed, there is emerging interest in the epigenetic regulation of aging, encompassing changes to histone dynamics and post-translational modifications, DNA methylation, and non-coding RNAs, among others [97]. Research in this area has yielded epigenetic clocks that effectively predict the age of a cell or tissue sample based on its pattern of DNA methylation [98]. Three recent studies probed the contribution of stochasticity to the predictive abilities of these clocks. Analysis of DNA methylation sequencing data from single cells [99] and bulk-sorted immune cells [100], along with the use of simulations and *C. elegans* transcriptomic data [101], found that a significant degree of predictive capacity in clocks can be explained by a stochastic element. These studies highlight the importance of further work in revealing how stochastic changes to epigenetic regulators outside of methylation interact with the aging process. The use of single-cell genomics technology, including single-cell ATAC-seq, will undoubtedly be a driving factor to propel this field forward.

Much work remains to be done in characterizing how stochastic events contribute to aging itself and differences in health outcomes during aging. As demonstrated here, as new areas of research in aging biology emerge, study of the stochastic components that interact and underlie these biological processes will be necessary. Mapping of the aging stochastome will undoubtedly provide greater insight into how aging affects biological systems at every level, in organisms from *C. elegans* to humans, paving the way for better control of health into late age.
